# Beyond Syncopal Episodes: A Complicated Hyperkalemic Emergency Manifesting As Complete Heart Block and Seizure Versus Convulsive Syncope Cascade

**DOI:** 10.7759/cureus.41473

**Published:** 2023-07-06

**Authors:** Mukosolu F Obi, Hyun Joon Cho, Manjari Sharma, Chelsea Noel, Paul R Gargiulo, Melissa M Vega, Win Hlaing

**Affiliations:** 1 Internal Medicine, Wyckoff Heights Medical Center, Brooklyn, USA; 2 School of Medicine, St. George's University, True Blue, GRD

**Keywords:** isoproterenol, head-up tilt test, seizure activity, convulsive syncope, complete heart block, refractory hyperkalemia, hyperkalemia induced ekg changes

## Abstract

We present a case involving an 87-year-old woman who had a hyperkalemic emergency. This condition was further complicated by complete heart block (CHB) and seizure-like activity. This case emphasizes the challenge of differentiating between seizures and convulsive syncope. Achieving an accurate diagnosis is essential for determining the appropriate medical treatment. This case report highlights the various symptoms and complications associated with hyperkalemia, emphasizing the importance of conducting a thorough examination to explore other potential causes. Additionally, it emphasizes the usefulness of the head-upright tilt test (HUTT) as a method to differentiate convulsive syncope from seizures, particularly in cases involving vagal stimulation.

## Introduction

Complete atrioventricular (AV) block, also known as third-degree AV block, is a kind of bradyarrhythmia that can lead to death if not detected and treated. Elderly people are more likely to develop AV node and Purkinje disease. Severe hyperkalemia is one of the possible causes of total AV block. Hyperkalemia, characterized by elevated potassium levels, disrupts the heart's electrical conduction system, leading to a complete heart block (CHB). Immediate intervention is essential to restore cardiac function and prevent complications. Convulsive syncope often seen with bradyarrhythmia, characterized by a sudden loss of consciousness and convulsive movements, poses challenges in differentiation from seizures due to overlapping symptoms. Precise differentiation between the two conditions is vital for tailored management. Hypoxemia, hyperkalemia, and hypothyroidism are all common metabolic reasons for AV block. Different quantities of high intracellular and low extracellular potassium ions are maintained by sodium potassium adenosine tri-phosphatase (Na-K ATPase) pumps, resulting in a resting membrane potential of -90 mV across the myocytes membrane [1]. As a result, an increase in the imbalance in cellular potassium homeostasis can have a negative influence on myocyte electrophysiology. Hyperkalemia has been linked to potentially fatal dysrhythmias such as ventricular tachycardia, ventricular fibrillation, idioventricular rhythms, and asystole. Complete AV block is most common in persons with pre-existing cardiac problems and is caused by severe hyperkalemia [1]. Convulsive syncope, on the other hand, is characterized by a brief loss of consciousness accompanied by convulsive movements. It is critical to distinguish between convulsive syncope and seizures since the underlying causes and therapeutic approaches differ greatly. It is critical to distinguish between the two to provide appropriate interventions and address the underlying reasons. This case study discussed a technique for distinguishing these symptoms and managing them.

## Case presentation

**Figure 1:**
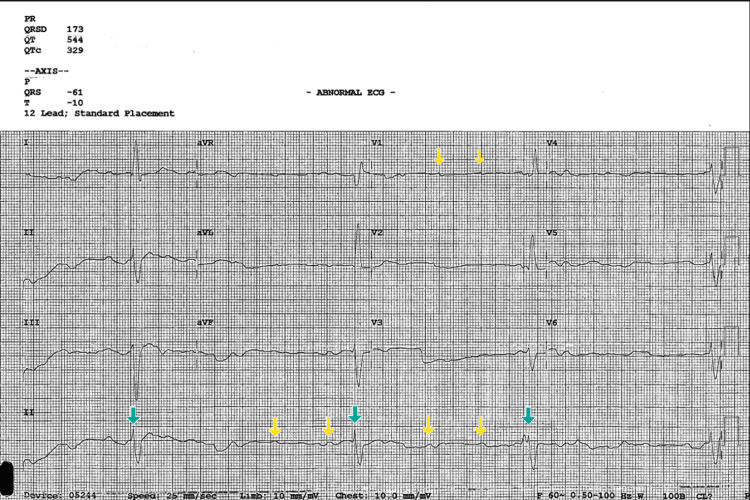
Initial EKG indicative of a third-degree CHB with an HR of 22 beats/min. The yellow arrows show multiple P waves with no conducting corresponding QRS, indicating an atrial rate independent of ventricular rate, a typical atrial ventricular dissociation seen in a third-degree heart block. The green arrow shows the QRS complex with no apparent P wave association

An 87-year-old female was brought to the ED with a complaint of experiencing repeated episodes of seizures. These seizures were accompanied by symptoms such as difficulty breathing and delayed thinking. The patient had a past medical history of type II diabetes, chronic obstructive pulmonary disease on a 2-liter home oxygen, obstructive sleep apnea, obesity hypoventilation syndrome (OHS), coronary artery disease with three stents, hypertension, diastolic heart failure, and chronic kidney disease stage III. Upon arrival in the ED, blood pressure (BP) was 124/56 mmHg and heart rate (HR) 50 beats/min, respiratory rate 24 breaths/min, and oxygen saturation of 90% on a 2-liter nasal cannula. The EMS reported that en route to the ED, the patient had multiple recurrent premature ventricular contractions on the telemonitor. The patient reported that she had four episodes of witnessed seizures by her daughter around early in the morning, associated with tonic-clonic movements of the upper and lower extremities bilaterally, diaphoresis, dyspnea, urinary and bowel incontinence, temporary lack of vision, 1-2 mins loss of consciousness, and a post-ictal confused state with a return to baseline between seizures. The patient denies chest pain, fever, chills, nausea, and vomiting and admits to mild dizziness and generalized weakness. Initial EKG was significant with HR 22 beats/min and complete AV dissociation, with independent atrial and ventricular rates seen in CHB (Figure 1).

At the time the initial EKG was taken, BP dropped to 90/54 mmHg and HR 20 beats/min. The review of biochemistry on initial encounter was significant for hyperkalemia and acute on chronic kidney injury (Table 1).

**Table 1:** Significant for persistent hyperkalemia after eight hours of hyperkalemia treatment. After hemodialysis, plasma potassium was corrected to the normal range. Acute kidney injury noted TSH: thyroid stimulating hormone, T4: thyroxine, GFR: glomerular filtration rate, CO2: carbon dioxide

Chemistry	Results
Urine toxicology	Negative
High-sensitivity troponin	53 ng/mL (normal range: 3-58.9 ng/L)
Calcium	9.1 mg/dL (normal range: 8.6-10.3 mg/dL)
Sodium	140 mEq/L (normal range: 136-145 mEq/L)
Potassium – Time 0	7.2 mEq/L (normal range: 3.5-5.2 mEq/L)
Potassium – Time 4 hours	6.9 mEq/L (normal range: 3.5-5.2 mEq/L)
Potassium – Time 8 hours	6.8 mEq/L (normal range: 3.5-5.2 mEq/L)
Potassium – After hemodialysis	4.2 mEq/L (normal range: 3.5-5.2 mEq/L)
Chloride	100 mmol/L (normal range: 96-106 mmol/L)
CO2	23 mEq/L (normal range: 23-29 mEq/L)
Blood urea nitrogen	50 mg/dL (normal range: 7-20 mg/dL)
GFR	21 (normal range: >60 ml/min/1.73m2)
Creatinine	2.17 mg/dL (normal range: 0.7-1.3 mg/dL)
Glucose	237 mmol/L (normal range: 70-100 mmol/L)
Lactic acid	2 mmol/L (normal range: 0-2 mmol/L)
Magnesium	2.2 mg/dL (normal range: 1.7-2.2 mg/dL)
Phosphorus	4.8 mg/dL (normal range: 3.4-4.5 mg/dL)
TSH	2.3 mIU/L (normal range: 0.5-5 mIU/L)
T4	1.0 µg/dL (normal range: 0.9-2.3 µg/dL)
Hemoglobin A1c	8.0% (normal range: 4-5.6%)

**Figure 2:**
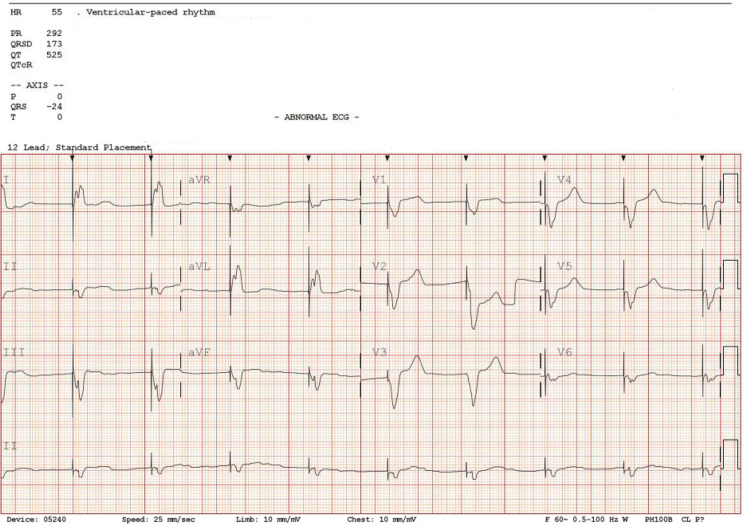
EKG after transvenous pacer, the black arrows showing ventricular paced rhythm with an HR of 55 beats/min

In the ED, the patient was started on calcium gluconate, a bolus injection of 10 units of regular insulin, followed by 50 mL of 50% dextrose, normal saline bolus with intravenous push of loop diuretics, and a total of 1.5mg of atropine. However, the bradycardia was refractory, and the patient remained hypotensive. A dopamine drip at 2 mcg/kg was started and titrated up to 5 mcg/kg for BP control. At this time, a cardiologist was consulted for intravenous pacemaker placement, and the patient was taken to the cardiac catheterization laboratory. A nephrologist was also consulted as there was no significant reduction in potassium level after the cocktail for hyperkalemia was given. Emergent hemodialysis was planned, and a left femoral double-lumen shiley central venous catheter was placed. A transvenous pacemaker (TVP) was placed at a rate of 60 beats/min, threshold at 10, and sensitivity at 2. The patient was then transferred to the ICU for closer monitoring. A nephrologist was consulted, and after further evaluation, given age, functional status, and comorbidities with acute presentation, renal replacement therapy was considered. The patient received a total of three hemodialysis sessions with repeat potassium of 4.2 mmol/l (Table 1). A repeat EKG was done after TVP (Figure 2).

**Figure 3:**
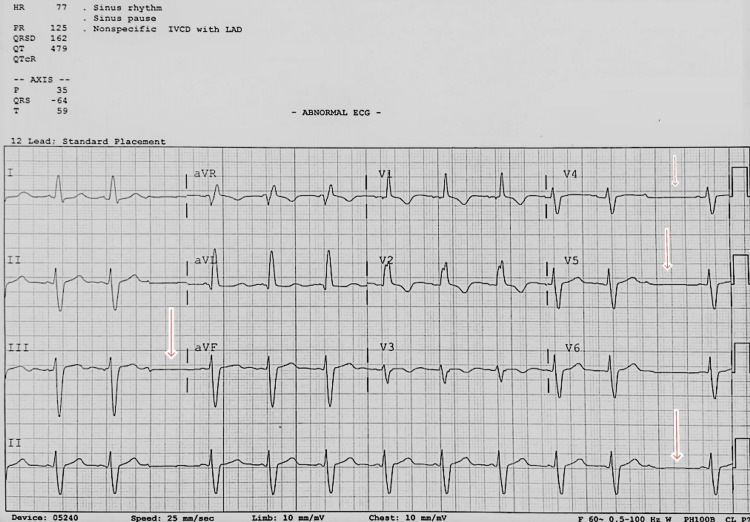
EKG, after the TVP was removed, showed an HR of 77 beats/min, left axis deviation, and intraventricular conduction delay. Red arrow showing non-conducting P waves

Echocardiogram showed a left ventricular ejection fraction of 55-60%, no wall motion abnormalities, and no profound valvopathy. A neurologist was consulted with a differential of seizures versus convulsive syncope. EEG showed normal long-term video monitoring in the awake and sleep states. The absence of abnormal findings on the EEG suggests that convulsive syncope, which occurs due to reduced blood flow to the brain in the presence of a CHB, is a more apparent explanation. No antiepileptic therapy was started, and closer monitoring was recommended with lorazepam 2 mg every two hours as needed. The patient was also placed on bilevel positive airway pressure at night due to obstructive sleep apnoea/OHS/chronic obstructive pulmonary disease to prevent CO2 narcosis which can cause altered mental status (Table 2). The cardiologist re-evaluated the patient and concluded on removing the TVP, and EKG was repeated (Figure 3).

**Table 2:** Arterial blood gas taken on room air indicative of respiratory acidosis pH: potential hydrogen, pCO2: partial pressure of carbon dioxide, pO2: partial pressure of oxygen, HCo3: bicarbonate

Arterial Blood Gas	Results
pH	7.33 (7.35-7.45)
pCO2	52 mmHg (35-45 mmHg)
pO2	83 mmHg (80-100 mmHg)
HCo3	27.4 mmol/L (25-27 mmol/L)

After showing improvement in the critical care unit, the patient was moved to a medical floor and subsequently discharged. The patient received instructions to schedule a follow-up appointment at the cardiology clinic and undergo a repeat EKG one week before the visit. The nephrologist discontinued hemodialysis and advised the patient and their family to steer clear of nephrotoxic substances while adhering to a renal diet.

## Discussion

Hyperkalemia, characterized by high levels of plasma potassium, can disrupt the electrical conduction pathway of cardiac myocytes. Among the components of the cardiac conduction system, the AV node is particularly sensitive to changes in serum potassium levels. This sensitivity is evident in the presence of peaked T waves, prolonged PR interval, and widened QRS complex on the EKG [1]. The severity of EKG changes correlates with the level of extracellular serum potassium, with levels above 7 mmol/L resulting in complete dissociation between atrial and ventricular rates, leading to bradyarrhythmia. In a normally functioning heart, electrical signals originate from the sinoatrial node, travel through the atria to the AV node, and are then transmitted through the bundle of His, Purkinje fibers, and ventricles. Hyperkalemia primarily impairs conduction in the Purkinje fibers and ventricles, although it can also cause AV block, albeit rarely, as reported in this case [1]. A CHB occurs when there is an impairment in the transmission of electrical signals from the supraventricular tissues to the ventricles, also known as sinoventricular propagation. Hyperkalemia-induced CHB is more commonly observed in patients with pre-existing heart disease. In the case of our patient, the presence of cardiac disease, specifically coronary artery disease, has already compromised the conduction system, making it more susceptible to external factors such as hyperkalemia.

In a patient experiencing hyperkalemia, the EKG initially shows peaked T waves, indicating synchronous shortening of action potentials and increased repolarization across the ventricular wall. Subsequently, the P wave widens, decreases in amplitude, and eventually disappears, while the QRS complex becomes wider. The sinus node, responsible for initiating the heart's electrical signals, is less affected by hyperkalemia due to its low resting potassium conductance. This is evident in its partially depolarized resting potential (ranging from -50 to -60 mV) and action potential, which relies on calcium currents. In contrast, the AV node, which serves as a bridge between the atria and ventricles, is more susceptible to the effects of hyperkalemia. This is because of the presence of adenosine-sensitive inward rectifier potassium channels (Kir3 family encoded by KCNJ3), which are sensitive to elevated potassium levels. The His-Purkinje tissue, responsible for rapid and efficient electrical conduction through the ventricles, has a higher resting potassium conductance compared to nodal tissues. As a result, its secondary pace-making capability is suppressed by hyperkalemia. In severe cases, this can lead to a complete cessation of electrical activity (asystole) [2].

Potassium ions play a vital role in maintaining the delicate balance between neuronal excitability and inhibition. Disruptions in this balance, as observed in cases of hyperkalemia, can lead to a shift toward persistent hyperexcitability, potentially triggering seizures. The hippocampus, known for its low seizure threshold, is often implicated in the origin or elaboration of complex partial (focal) epileptic seizures. Various studies have demonstrated that the hippocampus can be easily stimulated to generate recurrent seizures through the application of convulsant drugs or high-potassium (K+) solutions [3]. During a seizure episode in the hippocampus (ictal episode), large groups of neurons become excessively and synchronously active, leading to prolonged discharge lasting several seconds or minutes. In the periods between seizures (interictal periods), the epileptogenic focus in the hippocampus remains active, generating brief bursts of synchronized neuronal discharge that occur regularly or intermittently, lasting tens or hundreds of milliseconds [3]. Experimental data have provided evidence that an increase in extracellular potassium concentration can induce seizures and promote the frequency and propagation velocity of seizures [4].

Astrocytes, a type of glial cell found in the brain, play a vital role in supporting and maintaining the function of neurons. They are responsible for regulating the ionic balance and controlling the levels of extracellular potassium in the brain [4]. The concentration of potassium in the extracellular space is crucial for establishing the resting membrane potential of both neurons and astrocytes. During hyperkalemia, when there is an excessive amount of potassium in the bloodstream, astrocytes actively participate in modulating extracellular potassium levels. They achieve this by taking up the excess potassium ions through a process involving the sodium-potassium ATPase, which helps maintain overall homeostasis. The proper regulation of extracellular potassium is essential for normal neuronal activity. However, in the presence of hyperkalemia, the functioning of astrocytes can become impaired, resulting in an inadequate clearance of excess potassium. This disruption in potassium clearance by astrocytes can have a significant impact on the excitability and inhibition of neurons. Consequently, this imbalance in neuronal activity can contribute to the occurrence of seizures, as the abnormal electrical discharges associated with seizures are often linked to increased neuronal excitability [4].

Further research and investigations are required to better understand the mechanism by which hyperkalemia can cause seizures, particularly in patients with epileptic foci, and to determine how this knowledge can be applied to develop targeted treatments for this specific population. In this presented case, when the patient presented with hyperkalemia accompanied by tonic-clonic jerks and post-ictal confusion, the differential diagnosis primarily considered seizure activity due to hyperkalemia's potential to trigger seizures. This was contrasted with the possibility of convulsive syncope occurring in the context of bradyarrhythmia.

Even with point-based diagnostic criteria, it might be difficult to distinguish between syncope and seizures. The reports of non-medical witnesses are widely relied upon by physicians, and even when they are reliable, real syncope can still be mistaken for a seizure if anoxia-induced involuntary movements also occur. These motions include head turns, gaze deviations, automatisms, tonic spasms, and myoclonic jerks. A clinician who misdiagnoses syncope as epilepsy may fail to recognize significant arrhythmias such as sinus arrest, AV block, or ventricular tachycardia [5]. As presented in this case report, the patient had significant bradycardia and EKG was noted for CHB. According to the family, the patient exhibited seizure-like activity, including shaking, incontinence, and confusion. It is important to note that the patient had no previous history of seizures and was not taking any anti-seizure medication. Patients with arrhythmias may occasionally seek care from a neurologist due to various cerebral symptoms brought on by impaired blood flow to the brain. These symptoms can include sporadic episodes of dizziness and fainting, loss of consciousness (syncope), transient ischemic episodes, confusion, dementia, mental disorders (psychoses), and abnormal behavior mimicking other systemic metabolic imbalances like hypoglycemia. Furthermore, any condition leading to insufficient oxygen supply to the brain can cause a clear epileptic seizure [6].

A head-upright tilt test (HUTT) is a diagnostic procedure utilized to assess and distinguish individuals who experience convulsive syncope from epilepsy. Convulsive syncope refers to a sudden loss of consciousness accompanied by convulsive movements that resemble a seizure. This condition can be caused by either vasovagal-mediated hypotension or abnormal cardiac rhythm, resulting in a rapid decrease in BP. From a cardiac standpoint, the pathophysiology of convulsive syncope is likely associated with fatal arrhythmia, leading to reduced cerebral blood flow due to decreased stroke volume. In cases of vasovagal-induced convulsive syncope, the mechanism involves reduced activity of cardiac mechanoreceptors in response to decreased venous return, triggering sympathetic stimulation. This can cause forceful ventricular contractions, activating C fibers in the ventricles' base [7]. During sympathetic stimulation, there is a sudden surge in neural activity followed by a release of pressure on the baroreceptors, leading to vasodilation mediated by these receptors. Consequently, there is a further drop in BP, resulting in cerebral oxygen deprivation and subsequent loss of consciousness, sometimes accompanied by convulsive syncope. While this case presentation primarily focuses on cardiogenic-induced convulsive syncope, it is important to consider other potential causes for convulsive syncope to facilitate clinical differential diagnosis and increase awareness. Epilepsy, a neurological disorder characterized by abnormal neuronal signaling in the brain, can present with distinct features depending on the originating area in the brain. Epileptic seizures can be triggered by various factors such as stress, fatigue, or exposure to flashing lights. HUTT can provide valuable insights into differentiating between epilepsy and convulsive syncope, as they can manifest similar symptoms. By observing the absence of epileptic seizures during the test, healthcare professionals can gain important information for distinguishing between the two conditions.

A prospective non-randomized study was conducted to evaluate the effectiveness of HUTT in differentiating the diagnosis of convulsive syncope from epilepsy in patients experiencing idiopathic seizure-like episodes [7]. The study also incorporated the use of isoproterenol infusion to enhance the sensitivity of the test. The study included HUTT alone and HUTT with isoproterenol infusion, a medication that stimulates beta-adrenergic receptors, increasing HR and heart contractility. Isoproterenol enhances the test's sensitivity by provoking symptoms or fainting more likely in individuals with specific types of syncope. By mimicking real-life triggers through increased HR and cardiac workload, isoproterenol infusion helps uncover abnormal cardiac responses, reflexes, or autonomic dysregulation contributing to syncopal episodes. Findings showed that during baseline tilt, 40% experienced syncope with tonic-clonic seizure-like activity, while during isoproterenol infusion, 27% had similar experiences (total positive tests: 67%) [7]. EEG analysis revealed diffuse brain wave slowing, not characteristic of epileptic seizures, in all patients during convulsive episodes. The study concluded that combining HUTT with isoproterenol infusion is valuable in distinguishing vagally-induced convulsive syncope from epileptic seizures [7]. Of note, in our patient's case, HUTT was not performed, but considering the absence of seizure history, negative EEG results, and factors like AV nodal disease and sluggish vagal tone in the elderly, convulsive syncope is the most likely cause of seizure-like activity.

The degree of hyperkalemia and associated EKG changes can vary among patients, and significant clinical symptoms are typically observed in acute settings. Treatments for hyperkalemia are based on three strategies. If EKG changes are detected, intravenous calcium chloride or gluconate should be administered to stabilize the cardiac cell membrane. These medications block the adverse effects of extracellular potassium on cardiac myocytes by restoring the appropriate electrical gradient across the cell membrane. To decrease extracellular potassium levels, therapies that enhance potassium influx into the cells are employed. This can be achieved using beta-2 adrenergic agonists, sodium bicarbonate, and a combination of glucose and insulin [8]. Additionally, extracellular potassium can be eliminated from the body by using medications such as polystyrene sulfonate or through temporary hemodialysis [1]. In cases of hyperkalemia-induced CHB, emergent hemodialysis is recommended for a fast and effective outcome, aimed at reducing mortality. Temporary cardiac pacing is indicated in the presence of hyperkalemia or electrolyte toxicity-induced CHB. Different methods of pacing, such as transcutaneous pacing or transvenous pacing, can be used. However, transcutaneous pacing has certain limitations, including the inability to achieve capture and successfully pace the heart due to impediments from chest wall structures such as connective tissue, muscles, and bone, as well as difficulties in precisely locating the heart within the thorax and patient discomfort. Consequently, transvenous pacing is preferred as it is more comfortable and durable. Regarding the prospective study on convulsive syncope, treatment options involve the use of beta-adrenergic blocking agents, which can block vasovagal syncope by inhibiting ventricular contractions and mechanoreceptor activation. Transdermal scopolamine can decrease vagal tone, while disopyramide can increase peripheral vascular resistance [7].

## Conclusions

Prompt recognition and differentiation of hyperkalemia-induced CHB leading to convulsive syncope is crucial. Similar presenting symptoms make accurate diagnosis challenging based on patient history and physical examination. Careful evaluation of medical history, clinical presentation, and electrocardiographic findings aids in recognizing the etiology of CHB. Delays in treatment to lower extracellular potassium levels can increase morbidity and mortality. Hemodialysis is the fastest and most effective method for eliminating potassium in CHB patients unresponsive to guideline-directed treatment. A HUTT, especially with isoproterenol infusion, helps differentiate syncope from seizure with improved accuracy. Identifying CHB swiftly enables timely interventions to restore cardiac function while distinguishing convulsive syncope from seizures guides tailored treatment plans addressing the underlying cause. Collaboration among healthcare providers, including cardiologists, neurologists, and emergency medicine specialists, is critical for optimal patient outcomes in these challenging clinical scenarios.

## References

[1] Baratloo A, Haroutunian P, Rouhipour A, Safari S, Rahmati F (2015). Hyperkalemia-induced complete heart block. J Emerg Pract Trauma.

[2] Weiss JN, Qu Z, Shivkumar K (2017). Electrophysiology of hypokalemia and hyperkalemia. Circ Arrhythm Electrophysiol.

[3] Jensen MS, Yaari Y (1997). Role of intrinsic burst firing, potassium accumulation, and electrical coupling in the elevated potassium model of hippocampal epilepsy. J Neurophysiol.

[4] Bellot-Saez A, Kékesi O, Morley JW, Buskila Y (2017). Astrocytic modulation of neuronal excitability through K(+) spatial buffering. Neurosci Biobehav Rev.

[5] Zarraga IG, Ware DL (2007). Syncope, seizure, or both? An unusual case of complete heart block. J Electrocardiol.

[6] Schott GD, McLeod AA, Jewitt DE (1977). Cardiac arrhythmias that masquerade as epilepsy. Br Med J.

[7] Grubb BP, Gerard G, Roush K, Temesy-Armos P, Elliott L, Hahn H, Spann C (1991). Differentiation of convulsive syncope and epilepsy with head-up tilt testing. Ann Intern Med.

[8] Kosovali BD, Yildiz H (2018). Reversible complete atrioventriculer block in patient with mild hyperkalemia. J Cardiol Curr Res.

